# Mutagenicity evaluation to UV filters of benzophenone-6, benzophenone-8, and 4-methylbenzylidene camphor by Ames test

**DOI:** 10.1371/journal.pone.0255504

**Published:** 2021-09-02

**Authors:** Jing Zhang, Zhou-Tao Pei, Ya-Ni Zhao, Meng Zhang, Li-Ling Zhang, Wen-Qiang Wang, Jing-Ya Wu, Ran Yu, Li-Wei Sun

**Affiliations:** 1 School of Energy & Environment, Southeast University, Nanjing, Jiangsu, China; 2 Taihu Lake Water Environment Engineering Research Center (Wuxi), Southeast University, Wuxi, Jiangsu, China; 3 Water Pollution Control and Ecological Restoration Engineering Laboratory of Xizang, School of Information Engineering, Xizang Minzu University, Xianyang, China; VIT University, INDIA

## Abstract

Benzophenone (BPs) and 4-Methylbenzylidene Camphor are used as ultraviolet (UV) filters to protect the skin and hair in personal care products. The discharging of the three chemicals may endanger the receiving water ecosystem. In the present study, the mutagenicity of BP-6, BP-8, and 4-Methylbenzylidene Camphor was tested using the *Salmonella typhimurium* reverse mutation test (Ames test) in the system with and without rat liver microsomal preparations (S9). Four *S*.*typhimurium* strains, TA97, TA98, TA100, and TA102 were employed in the Ames tests. The mutagenicity was detected from all three chemicals. The addition of S9 increased the mutation ratios of three chemicals to four strains, except BP-6 to TA100 strain and 4-MBC to TA97 and TA98 strain. In the mixed experiment, all positive effects were detected in the absence of S9. However, the results all became negative in the presence of S9. For the mixture of BP-6 and 4-MBC, positive results were detected on four tester strains except for the TA100 strain. For the mixture of BP-6, BP-8, and 4-MBC, positive results were detected on four strains. The mixture test results showed antagonism in mutagenicity for the mixture of BP-6 and 4-MBC to TA98 and TA100 strains and the mixture of BP-6, BP-8, and 4-MBC to TA100 and TA102 strains.

## Introduction

In recent years, two types of ultraviolet (UV) filters, inorganic filters and organic filters, have been widely used in personal care products, such as sunscreen, lipstick, shampoo [1]. The content of UV filters in personal care products is generally between 0.1% -10% [1]. According to the regulations in the European Union and the United States, twenty-six and fourteen organic UV filters were permitted to be added to personal care products, respectively [2]. In Safety and Technical Standards for Cosmetics (2015 Edition) of China, 26 species of organic UV filters are allowed to be used in cosmetics [3]. Among UV filters, Benzophenone (BPs) and 4-Methylbenzylidene Camphor (4-MBC) are the most commonly used personal care products to protect the skin and hair from UV damage [4].

BPs and 4-MBC can be directly discharged to water bodies through human entertainment activities or sewage treatment plants with the cleaning and personal hygiene process [5]. Since the present treatment process cannot remove the BPs and 4-MBC effectively, BPs and 4-MBC have been consistently detected in natural water, sediment, soil, even tap water. For example, BP-3, BP-4, 4-MBC, and other BPs were detected in the Swiss River ranging from 6 to 68 ng/L [6]. BP-3 was detected in tap water in Spanish as high as 295 ng/L [7], and also the concentration of BP-4 was 36.6 ng/L [8]. Zhang *et al*. ‘s research results showed that the average concentration of BP-3 in the sediment of Songhua River in China was 0.38ng/g [9]. In addition, BPs were even detected in the marketed fish and human breast milk [10, 11].

BP-6, BP-8, and 4-MBC are three commonly used UV filters. In Senchez-Bunete’s research for the sediments, the detection frequency of BP-6 was the highest at the concentration of 1.2–6.1ng/L [12]. 4-MBC is also frequently detected in environmental water bodies and sewage treatment plants [7]. As one of the main biodegradation products of BP-3, BP-8 was reported to change the composition of human serum protein [13].

Although the detection concentrations of BPs and 4-MBC in the environment were low, they are chemically stable and not readily biodegradable [14]. Therefore, the BPs can gradually be enriched in the natural environment and be biomagnified through the food chain [15], forming persistent pollution, and bring potential risks to the ecological system and human health.

Some researches proved that BPs have acute toxicities to aquatic organisms [16–18]. Du Yang studied the acute toxicity of BP-1, BP-2, and BP-3 to *Chlorella vulgaris* and *Daphnia Magna*, they showed low to high toxicity to *C*. *Vulgaris* and *D*. *Magna* [19]. BPs were also reported inhibiting the development of the fertilized egg or alter gene expression [20, 21]. However, at present, there are few studies about the mutagenicity of BPs and 4-MBC.

The Ames test is a mature method, which uses histidine auxotrophic Salmonella strains to detect the mutagenicity of pollutants. It is an essential initial tool from the Safety and Technical Standards for Cosmetics. It is worldwide used to test the chemicals even the environmental samples for mutagenicity [22]. The assay is a required assay under Organization for Economic Co-operation and Development (OECD) guidelines for genetic toxicology testing [23, 24]. The test process is quick and the result is accurate [25].

Nakajima *et al*. studied the mutagenicity of BP and BP-1 by the Ames test employed the TA98 and TA100 strains [26]. Wen-Qian Wang *et al*. further studied the mutagenicity of BP and BP-1 by four TA strains and their mixing effects [27]. They got consistent results.

In the actual environment, pollutants usually exist in a mixed state. There is of significant importance to study the mixed toxicity of these chemicals. Ames test can exactly detect the comprehensive mutagenic effect of the mixture quickly and easily.

In this study, the mutagenicity of BP-6, BP-8, and 4-MBC was evaluated by the Ames test. Both the separated test and the different combined mixture of the three chemicals were carried out to study its mutagenic effects. The results of this study will reveal the genotoxicity of BPs series UV filters, comprehensively evaluate the toxicity of benzophenone series of compounds, and provide a more scientific basis for future ecological risk management in China.

## Materials and methods

### Chemicals

99% purity of BP-6 (CAS: 131-54-4), BP-8 (CAS: 131-53-3), and 4-MBC (CAS: 36861-47-9) were purchased from Aladdin Biochemical Technology Co., Ltd., (Shanghai, China). Analytical purity grade dimethyl sulfoxide (DMSO), used as a solvent, was obtained from Sinopharm Chemical Reagent Co., Ltd., (Shanghai, China). Rat liver microsomal preparations S9 was purchased from CHI Scientific, Inc. (Jiangsu, China).

### Test stains

Four histidine auxotrophic strains of *S*. *typhimurium*, TA97, TA98, TA100, and TA102 strain were employed. The strains were obtained from Jiangsu Provincial Center for Disease Control and Prevention and Guizhou Medical University, stored in an ultra-low temperature refrigerator at—80°C. TA97 and TA98 strain detect various frameshift mutagens while TA100 and TA102 strain detect mutagens that cause base-pair substitutions.

### Checking of the genotypes of tester strains

The genotypes of tester strains should be confirmed after receiving the cultures. The tests included histidine deficiency, lipopolysaccharide barrier defect, ampicillin resistance, UV sensitivity, tetracycline resistance, spontaneous reversion, and reversible characteristics, a diagnostic test. All tests were carried out according to the standard Ames bacterial mutagenicity test [25].

### Bacterial inhibition experiment of BP-6, BP-8, and 4-MBC

According to Safety and Technical Standards for Cosmetics, the maximum chemical dose should be 5000 μg/plate [3]. Therefore, the highest concentration of all tested chemicals was 5000 μg/plate, then diluted to 2500 μg/plate, 1000 μg/plate, 500 μg/plate, and 50 μg/plate to test the bacterial inhibition effects. BP-6, BP-8, and 4-MBC are all solved by DMSO. 0.1 ml of the above solution and 0.1 ml of bacterial at 1–2×10^9^ cells/mL solution per dish were added. Agar medium was used for the experiment.

### Ames test

The experiment was performed according to the guidelines recommended by the standard Ames bacterial mutagenicity test and Safety and Technical Standards for Cosmetics [3]. The test was carried out with and without the liver microsomal metabolic activation (S9) mixture, prepared by the standard method [27, 28]. Five doses, 0.5, 5, 50, 500, 2500 μg/plate, were set up for the tested chemicals. 0.1mL of *S*. *typhimurium* strains at 1–2×10^9^ cells/mL, 0.5mL of S9 mixture (system with S9) were added to the nutrient mediums. Three parallel plates were prepared for each tested chemicals dose and the negative control (blank and solvent) groups. All plates were incubated under the same conditions: 37 ± 0.5°C, 48 h, dark.

The blank and solvent control experiment was conducted to determine the normal spontaneous reversion rate and whether the solvent DMSO can cause mutagenicity on the tester strains. In the blank control, plates were poured with agar medium and 0.1mL bacterial. In the solvent DMSO control, 0.1mL bacterial and 0.1 mL DMSO, which is the highest dose, were added to the medium. The culture conditions were the same as the tested chemical group.

### Ames test of mixed chemicals

Ames tests of mixed chemicals were designed based on the results of each separated chemical experiment. The chemicals were mixed at a 1:1:1 ratio. According to the highest effective concentration in the system without S9 between the four strains. For BP-6 and 4-MBC, the highest concentration with a positive effect was both 50 μg/plate. For BP-8, the positive effect was 2500 μg/plate. Therefore, the highest mixed concentration of BP-6, BP-8, and 4-MBC were100% (50 μg/plate+2500 μg/plate +50 μg/plate). Then the mixtures were diluted in sequence to 50%, 10.0%, 1.0%, and 0.1%.

All tests were conducted with the same method as described for the separated chemicals.

### Data analysis and interpretation

The mutation ratio (MR) is calculated as

$$MR=X/X_{0}$$ (1)

X is the number of reverse mutation colonies on the chemical-treated plates, and X_0_ is the average colony number on the DMSO and blank control plates.

When the mutation ratio (MR) is ≥2 and the background is normal, the mutagenesis is positive [3].

All values are expressed as mean ± SD. Statistical analysis was performed with the Origin software (ver.2020).

## Results

### Negative control results

The number of spontaneous reverse mutation colonies in the blank and DMSO control was between 90–180 for TA97strain, 30–50 for TA98 strain, 100–200 for TA100 strain, and 240–320 for TA102 strain, which all qualified the quality control requirements of the Ames test [25, 29]. The results proved that the DMSO dose used in this study did not cause mutagenicity.

### Separated Ames test results

**Fig 1:**
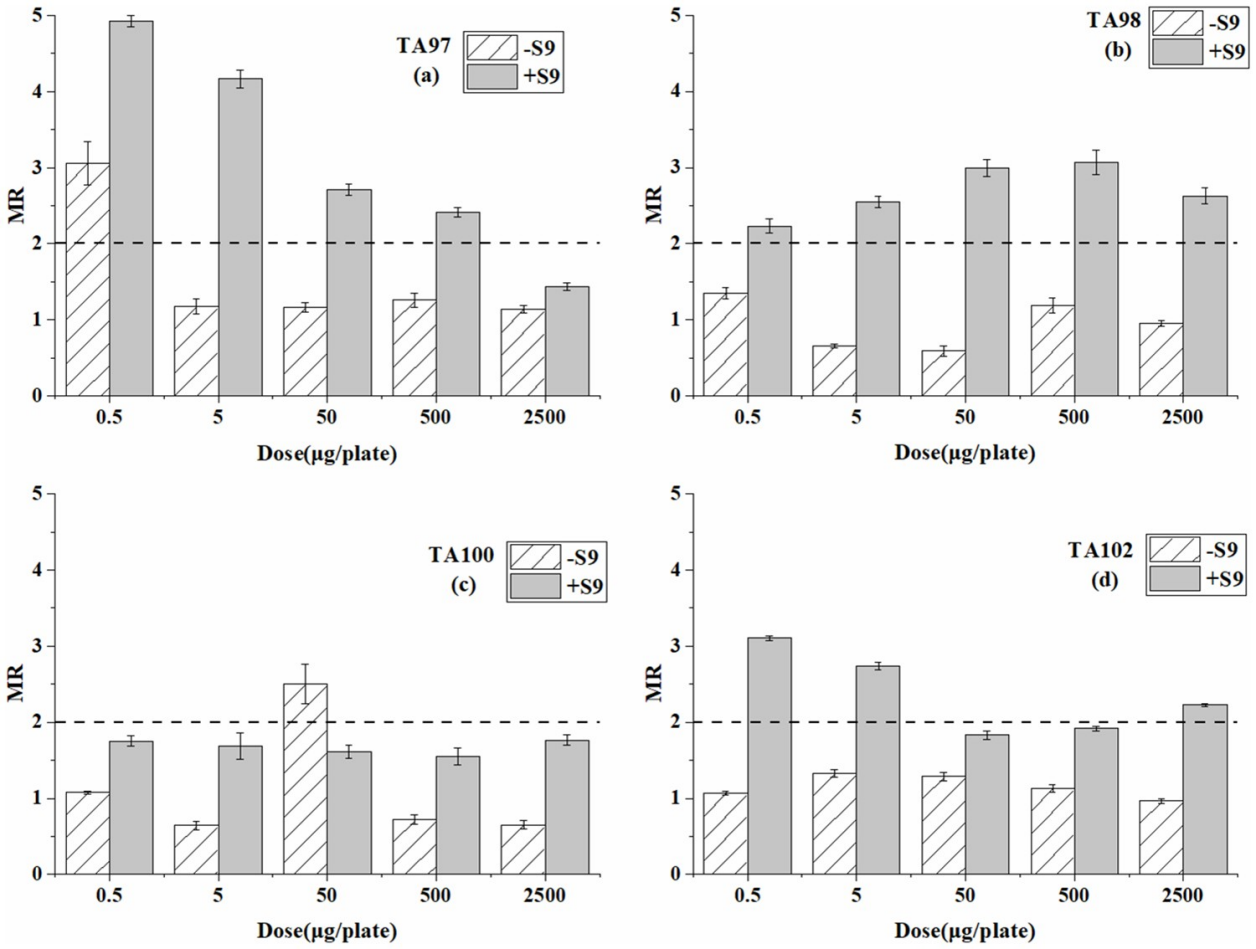
Mutagenesis of BP-6 to four strains in the presence and absence of S9 liver extract; (a) TA97 strain; (b) TA98 strain; (c) TA100 strain; (d) TA102 strain. The mutation ratio (MR) is the average ratio (±SE) from three parallel plates.

**Fig 2:**
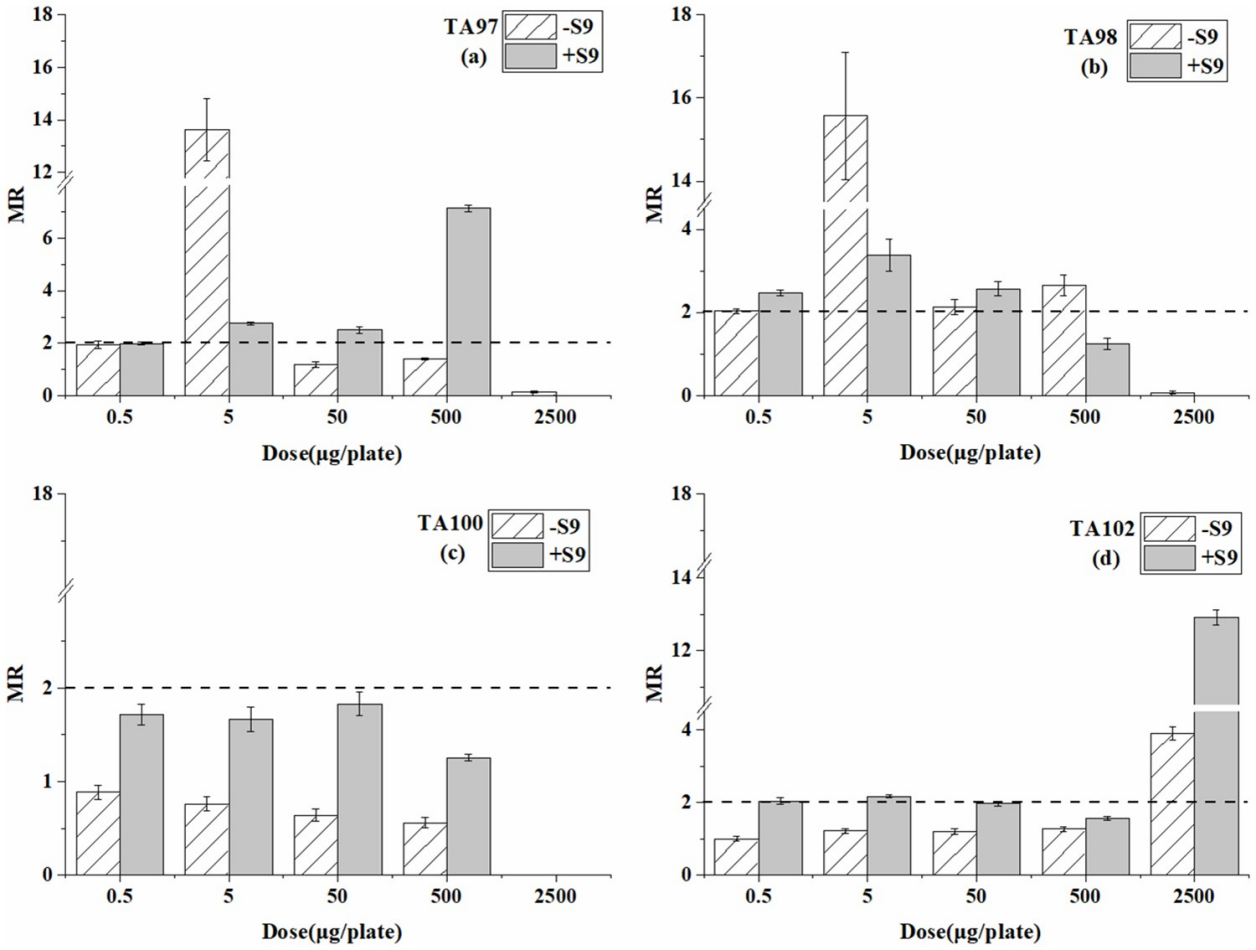
Mutagenesis of BP-8 to four strains in the presence and absence of S9 liver extract; (a) TA97 strain; (b) TA98 strain; (c) TA100 strain; (d) TA102 strain. The mutation ratio (MR) is the average ratio (±SE) from three parallel experiments.

**Fig 3:**
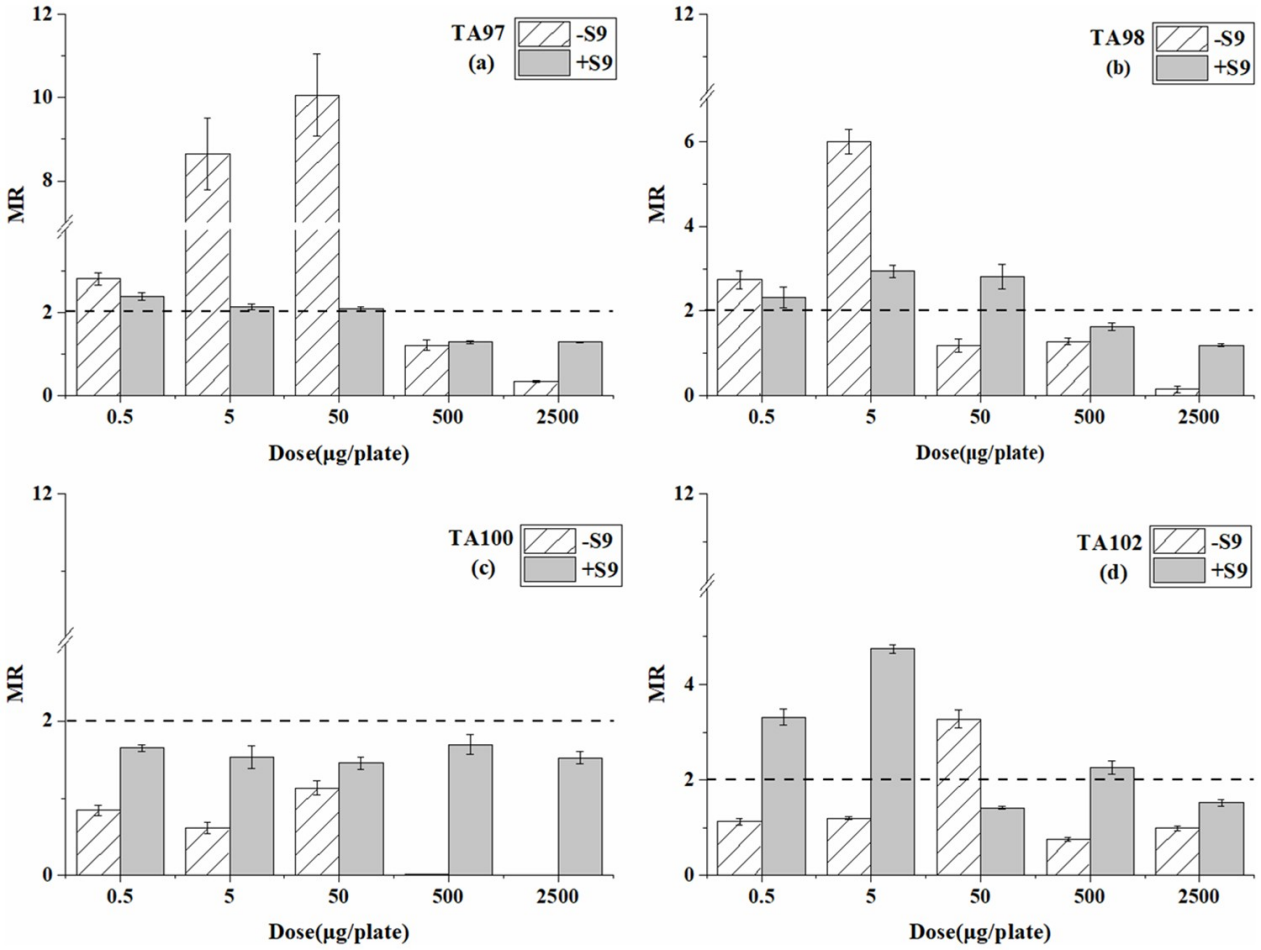
Mutagenesis of 4-MBC to four strains in the presence and absence of S9 liver extract; (a) TA97 strain; (b) TA98 strain; (c) TA100 strain; (d) TA102 strain. The mutation ratio (MR) is the average ratio (±SE) from three parallel experiments.

Tables 1–3 are the reverse mutation colonies of four *S*. *typhimurium* strains after exposure at different doses of BP-6, BP-8, and 4-MBC with and without S9 in the Ames test. The MR values at every dose are compared in Figs 1–3.

**Table 1:** Reverse mutation colonies of four *S*. *typhimurium* strains exposed to BP-6 with and without S9.

Chemicals	Dose (μg/plate)	TA97		TA98		TA100		TA102	
		-S9	+S9	-S9	+S9	-S9	+S9	-S9	+S9
DMSO	100	123±10	134±12	48±5	31±3	197±15	122±10	319±7	226±19
	0	168±13	156±9	33±4	34±3	184±5	143±4	289±7	226±9
BP-6	0.5	**446±42** *	**714±11** *	54±3	**73±3** *	206±4	233±9	325±6	**703±7** *
	5	172±14	**605±17** *	27±1	**83±3** *	123±11	223±23	404±14	**618±11** *
	50	170±9	**394±11** *	24±3	**98±4** *	**478±49** *	214±11	391±16	414±12
	500	184±13	**350±9** *	48±4	**100±5** *	137±12	205±15	344±14	433±7
	2500	166±7	209±8	39±2	**86±4** *	125±10	234±9	344±14	**503±5** *

* MR ≥ 2 compared to control.

**Table 2:** Reverse mutation colonies of four *S*. *typhimurium* strains exposed to BP-8 with and without S9.

Chemicals	Dose (μg/plate)	TA97		TA98		TA100		TA102	
		-S9	+S9	-S9	+S9	-S9	+S9	-S9	+S9
DMSO	100	123±10	134±12	48±5	31±3	197±15	122±10	319±7	226±19
	0	168±13	156±9	33±4	34±3	184±5	143±4	289±7	226±9
BP-8	0.5	283±20	288±8	**83±2** *	**81±2** *	169±15	228±14	304±21	**462±20** *
	5	**1989±172** *	**399±10** *	**631±62** *	**110±10** *	146±14	221±17	373±18	**490±9** *
	50	175±16	**362±18** *	**87±7** *	**84±6** *	123±12	242±17	367±20	448±14
	500	204±5	**1036±18** *	**107±10** *	41±5	107±10	167±4	387±20	354±13
	2500	22±3	0	3	0	0	0	**1192±55** *	**2919±45** *

* MR ≥ 2 compared to control.

**Table 3:** Reverse mutation colonies of four *S*. *typhimurium* strains exposed to 4-MBC with and without S9.

Chemicals	Dose (μg/plate)	TA97		TA98		TA100		TA102	
		-S9	+S9	-S9	+S9	-S9	+S9	-S9	+S9
DMSO	100	123±10	134±12	48±5	31±3	197±15	122±10	319±7	226±19
	0	168±13	156±9	33±4	34±3	184±5	143±4	289±7	226±9
4-MBC	0.5	**410±22** *	**346±13** *	**111±9** *	**75±8** *	161±13	219±6	342±23	**750±36** *
	5	**1264±125** *	**309±11** *	**243±12** *	**96±4** *	117±13	203±19	366±7	**1072±21** *
	50	**1469±142** *	**301±8** *	48±6	**92±9** *	217±18	193±11	**997±57** *	320±7
	500	177±18	187±5	52±3	53±3	4	225±17	231±12	**509±31** *
	2500	49±3	187±1	6	39±1	0	202±10	301±17	345±15

* MR ≥ 2 compared to control.

For the TA97 strain (Table 1 and Fig 1), the significant mutagenicity from BP-6 was detected only at the dose of 0.5 μg/plate without S9. While in the system with S9, the detected mutagenicity results were positive at each dose except the highest dose. For the TA98 and TA102 strains, the mutagenicity results were all negative without S9. However, when S9 was added, mutagenicity was detected at several doses. For the TA100 strain, weak mutagenicity was detected only at the dose of 50 μg/plate in the absence of S9. In the presence of S9, the results were all negative at every dose.

For the TA97 and TA102 strain (Table 2 and Fig 2), BP-8 showed mutagenicity only at one dose (5 μg/plate for TA97 strain and 2500 μg/plate for TA102 strain) without S9. When the S9 was added, mutagenicity was detected at several doses. For the TA98 strain, in the system with or without S9, positive mutagenicity was both detected. For the TA100 strain, no significant mutagenicity was detected at all concentrations with or without S9. The highest concentrations of BP-8 caused a strongly inhibited effect on the TA97, TA98, and TA100 strains.

For both TA97 and TA98 strain (Table 3 and Fig 3), the significant mutagenic effects were detected from 4-MBC both in the systems with and without S9. Furthermore, the reverse mutation colonies decreased in S9 added system. For the TA100 strain, no mutagenic activity was detected at all doses with and without S9, which was similar to BP-8. For the TA102 strain, significant mutagenicity was only detected at the dose of 50 μg/plate without S9. After adding S9, mutagenicity was also detected from the doses of 0.5, 5, and 500 μg/plate. The highest concentrations of 4-MBC inhibited on the TA97 and TA98 strain without S9.

### Mixed Ames test results

**Fig 4:**
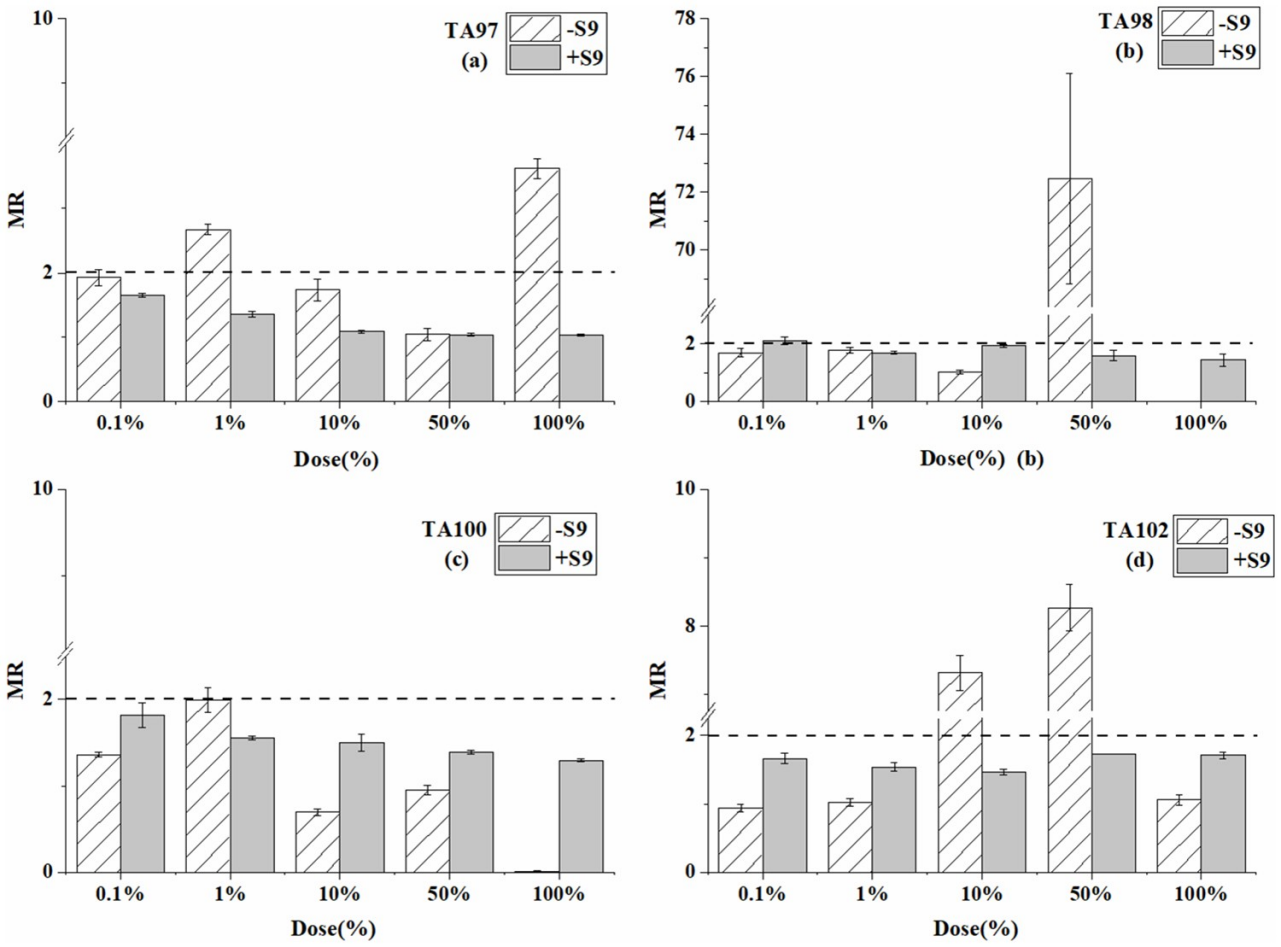
Mutagenesis of mixtures of BP-6 and 4-MBC to four strains in the presence and absence of S9 liver extract; (a) TA97 strain; (b) TA98 strain; (c) TA100 strain; (d) TA102 strain. The mutation ratio (MR) is the average ratio (±SE) from three parallel experiments.

**Fig 5:**
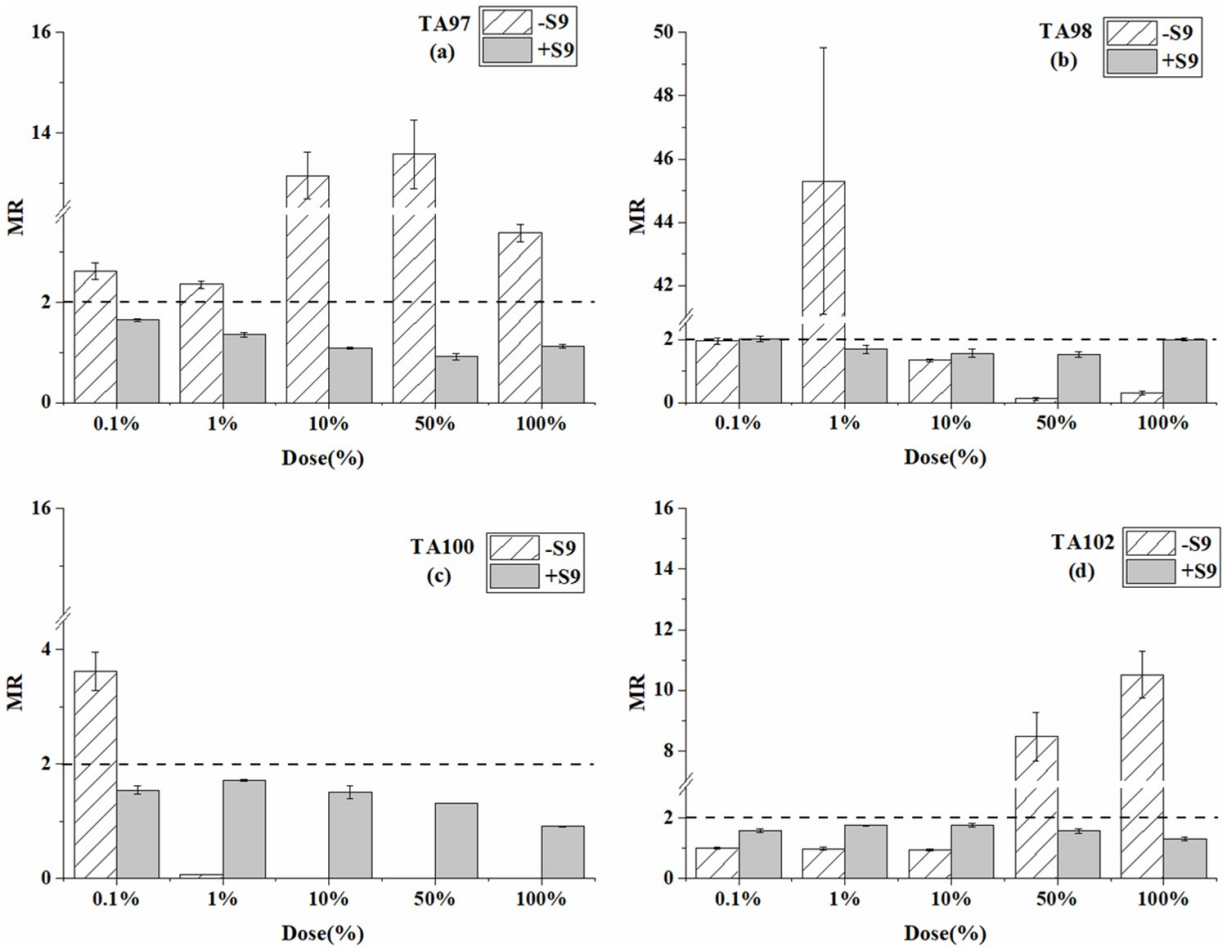
Mutagenesis of mixtures of BP-6, BP-8, and 4-MBC to four strains in the presence and absence of S9 liver extract; (a) TA97 strain; (b) TA98 strain; (c) TA100 strain; (d) TA102 strain. The mutation ratio (MR) is the average ratio (±SE) from three parallel experiments.

Tables 4 and 5 are the reverse mutation colonies of four *S*. *typhimurium* strains at different doses of the mixture of BP-6 and 4-MBC and the mixtures of BP-6, BP-8, and 4-MBC with and without S9. The MR values at every dose are compared in Figs 4 and 5.

**Table 4:** Reverse mutation colonies of four *S*. *typhimurium* strains exposed to mixtures of BP-6 and 4-MBC with and without S9.

Chemicals	Dose (percentage)	TA97		TA98		TA100		TA102	
		-S9	+S9	-S9	+S9	-S9	+S9	-S9	+S9
DMSO	100	123±10	134±12	48±5	31±3	197±15	122±10	319±7	226±19
	0	168±13	156±9	33±4	34±3	184±5	143±4	289±7	226±9
BP-6 + 4-MBC	0.10%	281±19	242±6	68±6	**70±4** *	260±5	245±8	284±16	373±13
	1.00%	**390±11** *	200±7	72±4	53±4	380±27	208±4	312±18	349±10
	10.00%	253±24	160±4	41±2	62±2	133±7	203±11	**2226±77** *	331±6
	50.00%	153±13	153±5	**2934±147** *	50±5	182±11	183±2	**2517±102** *	394±3
	100.00%	**528±22** *	153±3	0	47±5	2	175±5	323±24	391±11

* MR ≥ 2 compared to control.

**Table 5:** Reverse mutation colonies of four *S*. *typhimurium* strains exposed to mixtures of BP-6, BP-8, and 4-MBC with and without S9.

Chemicals	Dose (percentage)	TA97		TA98		TA100		TA102	
		-S9	+S9	-S9	+S9	-S9	+S9	-S9	+S9
DMSO	100	123±10	134±12	48±5	31±3	197±15	122±10	319±7	226±19
	0	168±13	156±9	33±4	34±3	184±5	143±4	289±7	226±9
BP-6+BP-8 + 4-MBC	0.10%	**383±24** *	240±4	78±4	63±5	**690±64** *	207±8	310±10	363±13
	1.00%	**344±11** *	195±6	**1834±170** *	53±4	13	233±8	301±16	400±7
	10.00%	**1920±69** *	160±4	54±2	50±3	0	201±10	287±11	395±10
	50.00%	**1981±99** *	131±8	5	48±4	0	175±2	**2577±246** *	356±12
	100.00%	**493±23** *	164±4	13	63±4	5	122±2	**3200±234** *	292±12

* MR ≥ 2 compared to control.

For the TA97 and TA102 strain (Table 4 and Fig 4), the MR value was ≥2 at two ratios without S9 (MR was 2.7 and 3.6 for TA 97 strain while 7.3 and 8.3 for TA100 strain). However, the mutagenicity was not detected when S9 was added. For the TA98 strain, mutagenicity was detected both in the two systems. For the TA100 strain, no mutagenicity was detected at each mixed ratio with and without S9. In conclusion, the mixture of BP-6 and 4-MBC caused positive mutagenicity on TA97, TA98, and TA102 strains in the absence of S9. The highest ratio of the mixture caused inhabitation effects on TA98 and TA100 strain without S9.

For the TA97 strain (Table 5 and Fig 5), each dose ratio of the mixture showed a positive effect without S9. For the TA98 and TA100 strain, the mixture caused a positive effect only at one ratio without S9 (1% for TA98 strain and 0.1% for TA100 strain). Especially, the MR value decreased sharply as the dose ratio increased. For TA102 strain, the mixture caused strong mutagenicity at 50% and 100% without S9 (MR value was up to10). Generally speaking, the mixture of BP-6, BP-8, and 4-MBC caused positive mutagenicity to all tested strains without S9. However, the mixture caused no mutagenicity on all four tested ratios when S9 was added. The high ratios of BP-6, BP-8, and 4-MBC mixture inhibited on TA98 and TA100 strain.

## Discussion

From the results, BP-6 caused mutagenicity on TA97 and TA100 strain, BP-8 and 4-MBC caused mutagenicity on TA97, TA98, and TA102 strain without S9. According to the Ames test standard, The TA97 and TA98 strain can detect the frameshift mutants, while the TA100 and TA102 strain detect DNA as a mutagenic substance for base-pair substitution [25]. Therefore, BP-6, BP-8, and 4-MBC all caused the two types of mutagenicity. Compared with our recent study of other BP series chemicals, BP caused mutagenicity only in the TA102 strain while BP-1 caused mutagenicity in TA97 and TA100 strain in the system without S9 [27]. So, the mutagenicity types of BP-6 and BP-8 were the same as BP-1, which caused the two types of mutagenicity. From the chemical structure, BP-1, BP-6, and BP-8 all have two benzene rings with substituents, while BP only contains two benzene rings without any substituents. Therefore, similar structures may cause similar mutagenicity types. Yamamoto studied the mutagenicity of BP-6, BP-8, and their chlorinated products using the TA98 and TA100 strains, they proved that the chlorinated products showed high mutagenicity [30].

In the separated experiment for 4-MBC with S9, the mutagenicity changes were complicated, the mutation rate decreased in TA97 and TA98 strain while it increased in the other two stains. For BP-6, the mutation ratio of TA97, TA98, and TA102 strain increased or changed from negative to positive. For BP-8, the mutagenicity also increased. In the two systems, the three chemicals all caused the two types of mutagenicity. In contrast with our results, the mutagenicity of BP and BP-1 for all strains was disappeared [27]. This may be because although BP-6, BP-8, and BP-1 all have substituents on the benzene ring, the types, quantities, and the position of substitution are different, so the metabolite after adding S9 may be different, showed different mutagenicity. Research reported that the mutagenicity of BP-6 and BP-8 changed after metabolism by microsomal fractions of hamsters [31], which was similar to our results.

In summary, the addition of S9 changed the mutation rates to all tester strains by the three tested chemicals but the mutagenicity types were not affected. These results indicated that the metabolite of the three chemicals increased the genetic toxicity risk, so the monitoring of the production and application processes of the three chemicals are worthy of vigilance.

In the mixed experiment after adding S9, the mutation rates were decreased by the addition of S9, which is contrary to the separated experimental results. No mutagenicity was detected except the mixture of BP-6 and 4-MBC for the TA98 strain. It probably due to a reaction that occurred between the chemicals themselves or metabolite products by S9. The mixed mutagenicity change results were similar to the study of BP and BP-1 mixture [27]. However, the results of this study cannot explain the actual mechanism. In the actual environment, BP-6, BP-8, and 4-MBC could be metabolized due to the microorganisms. Therefore, the mutagenicity may different from the laboratory results. In future studies, the joint toxicity mechanism of mixed chemicals should be strengthened.

The performances of the three chemicals in the mixtures were different from those in the separated experiments. In the mixture of BP-6 and 4-MBC, for the TA98 strain, BP-6 showed negative mutagenicity while 4-MBC itself showed positive mutagenicity at doses of 0.5 and 5 μg/plate in the separated test. However, in the mixed test, positive mutagenicity was observed when the dose of the mixture was 50%, in which the concentration of BP-6 and 4-MBC were both 25 μg/plate. From the results, it can be proved that, first, the mutagenicity in the mixture was from 4-MBC. Second, the corresponding dose of 4-MBC in the mixture was higher than that in the separate test, which indicated that the presence of BP-6 could reduce the mutagenicity of 4-MBC. For the TA100 strain, the result was similar to that of the TA98 strain. In the mixed experiment, there was no mutagenicity at every dose of the mixture of BP-6 and 4-MBC. However, a positive result was detected in BP-6 at 50 μg/plate. It could be considered that mutagenicity in the mixture was reduced.

From the result of BP-6, BP-8, and 4-MBC mixtures, for the TA100 strain, there are almost no visible strains observed on the plate at any ratio of the mixture, while in the separated experiment, positive results were observed from BP-6 at 5 μg/plate. The results showed that the mutagenicity after mixing is eliminated. Also, for TA102 strain, the effective concentration of BP-6, BP-8, and 4-MBC in the separated test is lower than that in the mixture test, which indicated that the mixing of BP-6, BP-8, and 4-MBC occurred antagonistic effect.

Furthermore, in the mixed test with S9, there is no mutagenicity at every ratio of the two mixtures compared to the system without S9. In our previous studies, no mutation was detected in four strains for both BP and BP-1 in the +S9 system, similar to the results in this study [27]. However, in the separated experiment with S9, mutagenicity was all detected for BP-6, BP-8, and 4-MBC. Generally speaking, the mixed test results indicated that BP-6 and 4-MBC showed antagonism in mutation for *S*. *typhimurium*, as well as BP-6, BP-8, and 4-MBC. The reason may be that the metabolic activity of S9 promoted the decomposition of the mixture. A chemical reaction occurred between the metabolites generated other chemicals which weakened the mutagenicity. From the previous and the present study, it can be proved that the mutagenicity of BP series chemicals depended on their chemical structure.

The acute toxicities of BP-6, BP-8, and 4-MBC to aquatic organisms were reported. The 48 h-LC_50_ of BP-6 and BP-8 on *D*. *Magna* were 12.79 and 2.17 mg/L, respectively [32]. According to the toxicity classification standard [33], BP-6 and BP-8 both caused medium-level and high-level toxicity to *D*. *Magna*. From the chemical structure, BP-8 has more hydroxyl groups on the benzene ring than BP-6, which may cause higher toxicity [32]. There were also reports about the toxicity of 4-MBC [34–36], the LC_50_ of 4-MBC on *D*. *Magna* was 0.8mg/L and the IC_10_ on *D*. *subspicatus* was 0.21mg/L, indicated high-level toxicity. In the present study, BP- 6, BP-8, and 4-MBC caused mutagenicity for four *S*. *typhimurium* strains. Therefore, the three chemicals showed potential environmental risks and needed to be noted.

For the mixed test, BP-6 and 4-MBC mixture showed antagonism effect in mutagenicity for *S*. *typhimurium* strains, as well as BP-6, BP-8, and 4-MBC mixture. Wen-Qian Wang *et al*. have studied the mutagenicity of the mixture of BP, BP-1, the result was similar to our study [27]. In the previous research, the eco-toxicity of the mixture of BP-3 and BP-4 was studied, showed antagonism to *C*. *vulgaris* (algae), *D*. *Magna* (zooplankton), and *Brachydanio rerio* (fish) [19]. Those results about the UV filters mixture are consistent with the present results. The mixing of UV filter chemicals in the environment is more diversified, and its toxicity effects are predicted to be more complicated.

Before conducting the Ames test, bacterial growth inhibition tests were carried out. The test dose was 5000 μg/plate for all three tested chemicals, no strain growth was observed. The results of the separated Ames tests showed that when the dose was higher than 500 μg/plate, there were growth-inhibiting effects on TA98 and TA100 by BP-8, 4-MBC. Also, the highest concentration of the two mixtures in the experiment caused inhibiting effects on TA98 and TA100 strain. There are two possible reasons: first, the tested chemicals may destroy the cell membrane of TA98 strain and TA100 strain, make the intracellular lysate outflow, and DNA exudate, thereby inhibited the growth of the two strains. Second, the high dose of the tested chemicals may cause DNA damage which is irreversible [37].

Although the research results showed that the concentrations of BPs and 4-MBC in the environment are low [14], the survey results of the actual measured concentrations of the environment are limited, which makes the evaluation of the ecological risks about BPs and 4-MBC difficult. The present study and our previous studies evaluated the acute toxic effects and mutagenicity of some BP series UV filters, contributed to their ecological risks assessment. However, further research should be conducted to study the toxic effects of other more BPs, forming a systematic toxicity database and comprehensive assessment about these chemicals.

## Conclusions

1. In the separated experiment, BP-6, BP-8, and 4-MBC caused mutagenicity to the tester strains in the system without S9. After S9 was added, the mutation rates were increased by three chemicals to four strains except BP-6 to TA100 strain and 4-MBC to TA97 and TA98 strain.
2. In the mixed experiment, the mixture of BP-6 and 4-MBC and the mixture of BP-6, BP-8, and 4-MBC caused positive mutagenicity without S9. When S9 was added, the mutation rates were decreased. The mixture of BP-6 and 4-MBC showed antagonism in mutagenicity for TA98 and TA100 strains and the mixture of BP-6, BP-8 and 4-MBC also showed antagonism in mutagenicity for TA100and TA102 strains.
3. The addition of S9 caused different effects in the separated experiment and mixed experiment. The mutation rates increased in the separated experiment while they decreased in the mixed experiment. Therefore, the mechanism of the mixing effects and the change of mutagenicity after S9 metabolism needs further study.

## Supporting information

S1 File Data and results. (XLSX)

## Abbreviations

The following abbreviations are used in this manuscript:

4-MBC: 4-Methylbenzylidene Camphor
BP-6: Benzophenone-6
BP-8: benzophenone-8
